# Synergistic effect of lipoprotein(a) and high-sensitivity C-reactive protein on the risk of all-cause and cardiovascular death in patients with acute myocardial infarction: a large prospective cohort study

**DOI:** 10.3389/fendo.2024.1392859

**Published:** 2024-05-15

**Authors:** Zhenwei Wang, Junnan Tang, Qian Shi, Lijuan Fang, Naifeng Liu, Jinying Zhang

**Affiliations:** ^1^ Department of Cardiology, The First Affiliated Hospital of Zhengzhou University, Zhengzhou, China; ^2^ Key Laboratory of Cardiac Injury and Repair of Henan Province, Zhengzhou, China; ^3^ Henan Province Clinical Research Center for Cardiovascular Diseases, Zhengzhou, China; ^4^ Neonatal Intensive Care Unit, The Third Affiliated Hospital of Zhengzhou University, Zhengzhou, China; ^5^ Department of Cardiology, The First Hospital of Hohhot, Hohhot, China; ^6^ Department of Cardiology, Zhongda Hospital, School of Medicine, Southeast University, Nanjing, China

**Keywords:** high-sensitivity C-reactive protein, all-cause death, cardiovascular death, synergistic effect, lipoprotein(a)

## Abstract

**Objective:**

Although lipoprotein(a) [Lp(a)] and high-sensitivity C-reactive protein (Hs-CRP) are closely associated with the mortality of acute myocardial infarction (AMI), their synergistic effect on the risk of death remains unknown. Therefore, this study aimed to explore the combined effect of Lp(a) and Hs-CRP on the incidence of all-cause and cardiovascular death in AMI patients.

**Methods:**

A comprehensive cohort study enrolled 912 AMI patients, categorizing them into four groups based on Lp(a) and Hs-CRP levels: Group 1 [Lp(a) < 30 mg/dL & Hs-CRP < 2 mg/L], Group 2 [Lp(a) < 30 mg/dL & Hs-CRP ≥ 2 mg/L], Group 3 [Lp(a) ≥ 30 mg/dL & Hs-CRP < 2 mg/L], and Group 4 [Lp(a) ≥ 30 mg/dL & Hs-CRP ≥ 2 mg/L]. Cox regression analysis, Kaplan-Meier survival analysis and sensitivity analysis were employed to determine the combined effects of Lp(a) and Hs-CRP on the risk of all-cause and cardiovascular death.

**Results:**

Over a median observation period of 38.98 months, 217 patients passed away, with 137 deaths attributed to cardiovascular causes. The multivariate Cox regression analysis revealed that in the comprehensively adjusted Model 3, only Lp(a) and the combination of Lp(a) and Hs-CRP exhibited a strong association with cardiovascular death risk. Specifically, for Lp(a) levels ≥ 30 mg/dL compared to < 30 mg/dL, the hazard ratio (HR) was 2.434 with a 95% confidence interval (CI) of 1.653–3.583 (P < 0.001); for log_10_(Lp(a)), the HR was 2.630 with a 95% CI of 1.530–4.523 (P < 0.001); for Group 4 versus Group 1, the HR was 2.346 with a 95% CI of 1.054–5.220 (P = 0.037); and for Group 4 versus Groups 1 + 2 + 3, the HR was 1.878 with a 95% CI of 1.284–2.748 (P = 0.001). Sensitivity analysis indicated that the synergy between Lp(a) and Hs-CRP continued to be independently associated with the risk of cardiovascular death. For Group 3 versus Group 1, the HR was 3.353 with a 95% CI of 1.133–9.917 (P = 0.029); for Group 4 versus Group 1, the HR was 3.710 with a 95% CI of 1.466–9.392 (P = 0.006); and for Group 4 versus Groups 1 + 2 + 3, the HR was 2.433 with a 95% CI of 1.620–3.656 (P < 0.001).

**Conclusions:**

Compared to elevated levels of either Lp(a) or Hs-CRP alone, the concurrent high levels of both significantly increased the risk of cardiovascular death in patients with AMI, underscoring the importance of considering their combined effects in the prognostic management of AMI patients.

## 1 Introduction

Cardiovascular disease (CVD) remains a significant global public health issue. Data from the Global Burden of Disease (GBD) Study 2019 indicate that over the last 30 years, global CVD incidence and mortality have increased by 93.0% and 53.7%, respectively. Ischemic heart disease (IHD) cases and related deaths rose to 197 million and 9.14 million in 2019 (1). In China, IHD incidence and mortality rates were 246.06 and 131.75 per 100,000 people, respectively, that year (2). Acute myocardial infarction (AMI), a leading cause of death among IHD sufferers, contributes to around 7 million deaths annually, highlighting the need to address controllable AMI risk factors to reduce mortality (3). Despite managing traditional risk factors like hypertension, diabetes, and low-density lipoprotein cholesterol (LDL-C), high AMI mortality rates suggest the presence of other unaddressed cardiovascular risks (4–6).

Lipoprotein(a) [Lp(a)], a complex LDL-like particle with genetic regulation, is linked to CVD risk due to its components such as apolipoprotein(a) [apo(a)], apolipoprotein B-100, cholesterol, and its homology with plasminogen, which contribute to oxidative stress, atherosclerosis, calcification, thrombosis, and inflammation (4, 7, 8). Despite varying levels across ethnicities, elevated Lp(a) is causally associated with atherosclerotic CVD (8).Furthermore, high-sensitivity C-reactive protein (Hs-CRP), a marker indicating systemic inflammation, also correlates with increased risks of all-cause and cardiovascular mortality (4, 9–14), underscoring the importance of monitoring Hs-CRP levels in various populations to improve cardiovascular outcomes.

While the individual effects of Lp(a) and Hs-CRP on mortality are known, their combined impact on mortality in AMI patients remains unclear. This study explored their synergistic effects on all-cause and cardiovascular death in AMI patients, aiming to advance integrated risk management strategies for high-risk populations.

## 2 Materials and methods

### 2.1 Study population

**Figure 1:**
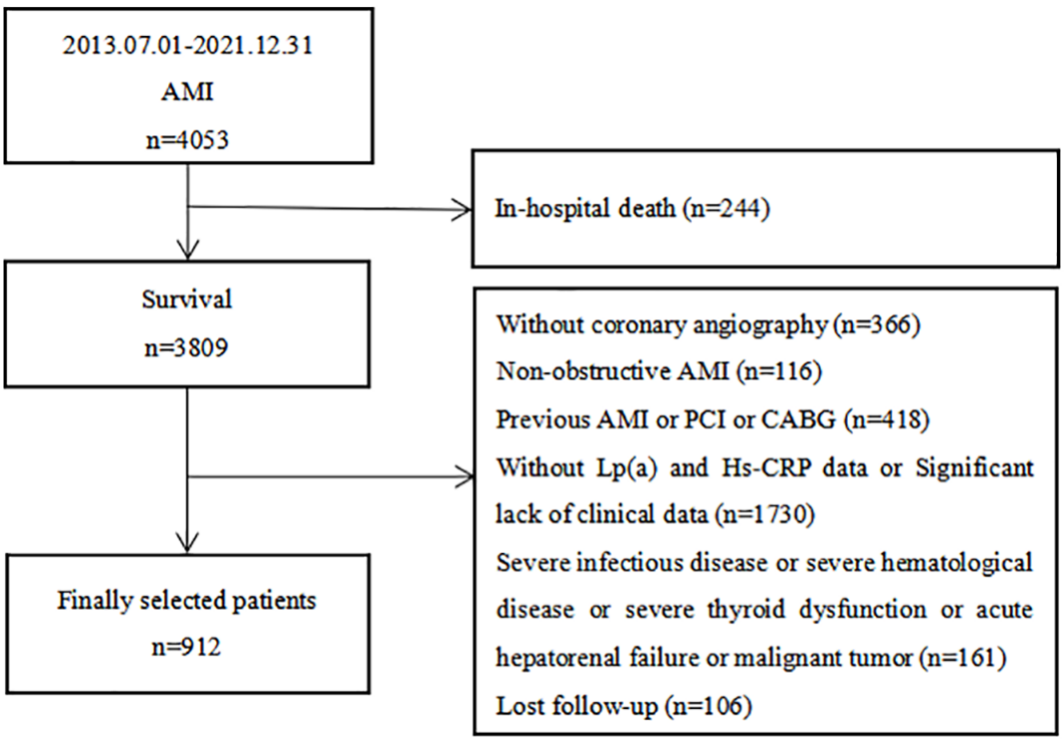
Flow chart of the study population. AMI, acute myocardial infarction; PCI, percutaneous coronary intervention; CABG, coronary artery bypass graft; Lp(a), lipoprotein (a); Hs-CRP, high-sensitivity C-reactive protein.

This comprehensive prospective cohort study enrolled 912 AMI patients who were admitted to the Department of Cardiology at Zhongda Hospital, which is affiliated with Southeast University. These patients underwent coronary angiography between July 1, 2013, and December 31, 2021 (**Figure 1**). The inclusion criteria included: (1) a confirmed diagnosis of AMI and (2) being age ≥ 18 years at the time of admission. The exclusion criteria included: (1) absence of coronary angiography; (2) a diagnosis of non-obstructive AMI; (3) a history of previous AMI, percutaneous coronary intervention (PCI), or coronary artery bypass graft (CABG); (4) the presence of severe infectious or hematological diseases, significant thyroid dysfunction, acute hepatorenal failure, or malignant tumors; (5) in-hospital death; (6) missing data on Lp(a) and Hs-CRP levels or a significant lack of other clinical data; and (7) loss to follow-up. The protocol received approval from the Clinical Research Ethics Committee of Zhongda Hospital affiliated to Southeast University, adhering to the ethical guidelines of the Declaration of Helsinki. All participants provided informed consent before their inclusion in the study.

### 2.2 Data collection and definitions

In this comprehensive study, we meticulously collected and analyzed a wide array of variables to understand their impact on AMI outcomes. Demographic variables included age, sex, smoking status, and a family history of coronary heart disease (CHD). We also considered a range of comorbid conditions such as diabetes, hypertension, hyperlipidemia, stroke, and chronic kidney disease (CKD), alongside previous medication usage including hypotensive drugs, hypoglycemic drugs, and lipid-lowering drugs.

Key biomarker variables assessed included: left ventricular ejection fraction (LVEF), body mass index (BMI), systolic and diastolic blood pressure (SBP/DBP), heart rate, white blood cell count (WBC), hemoglobin, platelet count, albumin, lipid profile (triglycerides, total cholesterol [TC], LDL-C, high-density lipoprotein cholesterol [HDL-C], apolipoprotein A1 [ApoA1] and B [ApoB], Lp(a), uric acid, fasting blood glucose (FBG), hemoglobin A1c (HbA1c), fibrinogen (FIB), and Hs-CRP.

Variables related to coronary angiography included: the presence of left main disease, three-vessel disease, multiple vessel disease, the number of diseased vessels, Gensini score, procedures performed (PCI/CABG), and specifics of stent deployment (number and length). Discharge medications recorded encompassed:: aspirin, clopidogrel, tegretol, statins, proprotein convertase subtilisin/kexin type 9 inhibitors (PCSK9i), beta-blockers, angiotensin converting enzyme inhibitors (ACEI), angiotensin receptor blocker (ARB), calcium channel blocker (CCB), insulin, and oral hypoglycemic agents.

Definitions for our study variables were aligned with established clinical criteria. Smoking was defined as having any regular smoking history, irrespective of current status. Diabetes was defined by FBG ≥ 7.0 mmol/L, random blood glucose ≥ 11.1 mmol/L, HbA1c ≥ 6.5%, or a diabetes diagnosis (15). Hypertension was identified by SBP/DBP ≥ 140/90 mmHg on multiple measurements or a history of hypertension (16). Hyperlipidemia criteria were fasting TC ≥ 5.72 mmol/L or triglycerides ≥ 1.70 mmol/L (17). Stroke was defined as a history of a previous stroke diagnosis or a stroke definitively diagnosed during this hospitalization, including ischaemic and haemorrhagic types. CKD was defined as a decline in renal function characterized by an estimated glomerular filtration rate (eGFR) < 60 ml/min/1.73m^2^, the presence of a marker of renal damage, or both, persisting for at least 3 months, regardless of the cause (18). The eGFR was calculated according to a modified version of the Modification of Diet in Renal Disease formula adapted to the Chinese population (19). The diagnosis of AMI, including ST-segment elevafion myocardial infarction (STEMI) and non-ST-segment elevation myocardial infarction (NSTEMI) subtypes, was based on clinical, electrocardiographic, laboratory, and angiographic criteria. The Killip class was categorized into class I or ≥ II. The GRACE score was calculated according to the Global Registry of Acute Coronary Events system (20). The Gensini score was employed to evaluate coronary lesion severity (21). Definitions for left main disease, multiple vessel disease, and three-vessel disease were based on the degree of stenosis observed in coronary angiography.

### 2.3 Measurement of Lp(a) and Hs-CRP

In our study, the concentrations of circulating Lp(a) and Hs-CRP were quantified using an immunoturbidimetric assay. This method involves the binding of Lp(a) or Hs-CRP in the sample to specific anti-human antibodies within the reagent, forming an antigen-antibody immune complex. This complexation causes turbidity in the sample, which is directly proportional to the concentration of Lp(a) or Hs-CRP present. The normal reference ranges established for Lp(a) and Hs-CRP are 10–30 mg/dL and 0–2 mg/L, respectively.

### 2.4 Follow-up and outcomes

Follow-up extended from the date of discharge until either the occurrence of death or December 31, 2022. We utilized a combination of outpatient visits, telephone interviews, and re-hospitalization records to gather follow-up data. The primary outcomes of interest were all-cause death and cardiovascular death. All-cause death was defined as death from any cause, encompassing both cardiovascular and non-cardiovascular causes. Cardiovascular death was specifically attributed to cardiac events such as AMI, heart failure, malignant arrhythmias, and deaths from unspecified noncardiac origins.

### 2.5 Statistical analysis

The analysis of categorical variables, expressed as counts (percentages), was performed using either the chi-square test or Fisher’s exact test to identify differences between groups. For continuous variables following a normal distribution, expressed as mean ± standard deviation, we employed the independent samples T-test or one-way ANOVA for between-group comparisons. For variables with a skewed distribution, expressed as median (interquartile range), the Mann-Whitney U or Kruskal-Wallis H test was utilized. Given the skewed nature of Lp(a) and Hs-CRP levels (**Supplementary Figure S1**), log_10_ transformation was applied to facilitate regression analysis. Univariate Cox regression analysis explored the relationship of all variables with the risks of all-cause and cardiovascular death. Multivariate Cox regression and Kaplan-Meier survival analyses further assessed the impact of Lp(a), Hs-CRP, and their combined effect on death risks. Sensitivity analysis was conducted to evaluate the stability of these associations, particularly after excluding patients with an eGFR < 30 ml/min/1.73m^2^. Statistical computations were carried out using SPSS 26.0, R 4.1.3, and GraphPad Prism 8.0, with a two-tailed P value of < 0.05 denoting statistical significance.

## 3 Results

### 3.1 Baseline characteristics

**Supplementary Table S1** presented the baseline characteristics of the 912 patients enrolled in this study, with an average age of 64.66 years, and 77.7% being male. Over a median follow-up of 38.98 months, 217 patients passed away, including 137 from CVD. The group experiencing all-cause death was characterized by older age, a higher prevalence of diabetes, hypertension, stroke, and CKD, increased usage of hypotensive and hypoglycemic drugs, elevated SBP, Lp(a), uric acid, HbA1c, FIB, Hs-CRP, and GRACE scores. This group also had fewer males and smokers, a lower incidence of hyperlipidemia, reduced STEMI occurrences, fewer patients with Killip class ≥ II, and lower levels of LVEF, BMI, DBP, hemoglobin, albumin, triglycerides, ApoA1, and eGFR (P < 0.05). Coronary angiographic data revealed that the all-cause death group had a higher incidence of three-vessel and multiple vessel diseases, more diseased vessels, and higher Gensini scores, but underwent PCI/CABG less frequently and had shorter stent lengths (P < 0.05). Regarding discharge medications, this group was more likely to be prescribed clopidogrel, CCB, and insulin, but less likely to receive ticagrelor and PCSK9i (P < 0.05). Comparatively, the cardiovascular death group shared similar characteristics with the all-cause death group, including older age and higher rates of comorbidities, medication use, and specific biomarkers. Notably, this group had higher LDL-C and ApoB levels. Coronary angiographic findings and discharge medication patterns also followed a similar trend, with higher incidences of severe coronary artery diseases and different medication usage (P < 0.05).

**Tables 1**–**3** and **Figure 2** organized patients into four groups based on their Lp(a) and Hs-CRP levels: Group 1 [Lp(a) < 30 mg/dL & Hs-CRP < 2 mg/L], Group 2 [Lp(a) < 30 mg/dL & Hs-CRP ≥ 2 mg/L], Group 3 [Lp(a) ≥ 30 mg/dL & Hs-CRP < 2 mg/L], and Group 4 [Lp(a) ≥ 30 mg/dL & Hs-CRP ≥ 2 mg/L]. Significant differences in both all-cause and cardiovascular death rates were observed across these groups (P < 0.001), with Group 4 showing the highest death rates (**Table 3**). Specifically, Group 4 had elevated all-cause and cardiovascular death rates compared to the other groups (P < 0.05), while Group 2 had increased all-cause death rates and Group 3 had higher cardiovascular death rates than Group 1 (P < 0.01) (**Figure 2**). Other variables also significantly differed among the groups (P < 0.05) (**Tables 1**, **2**).

**Table 1:** General information of patients stratified by Lp(a) and Hs-CRP categories.

	Group 1	Group 2	Group 3	Group 4	P value
N	164	361	78	309	
Age, years	61.54 ± 12.33	64.43 ± 13.56	64.78 ± 13.49	66.54 ± 12.86	0.001
Sex, male, n (%)	134 (81.70%)	278 (77.00%)	51 (65.40%)	246 (79.60%)	0.028
Smoking, n (%)	96 (58.50%)	202 (56.00%)	34 (43.60%)	164 (53.10%)	0.146
Family history of CHD, n (%)	16 (9.80%)	39 (10.80%)	6 (7.70%)	49 (15.90%)	0.073
Comorbidities, n (%)					
Diabetes	52 (31.70%)	136 (37.70%)	27 (34.60%)	123 (39.80%)	0.352
Hypertension	111 (67.70%)	254 (70.40%)	53 (67.90%)	243 (78.60%)	0.025
Hyperlipidemia	73 (44.50%)	140 (38.80%)	34 (44.20%)	139 (45.10%)	0.352
Stroke	35 (21.30%)	85 (23.50%)	19 (24.40%)	89 (28.80%)	0.264
CKD	14 (8.50%)	81 (22.40%)	13 (16.70%)	96 (31.10%)	< 0.001
Treatment, n (%)					
Hypotensive drugs	80 (48.80%)	194 (53.70%)	41 (52.60%)	173 (56.00%)	0.518
Hypoglycemic drugs	38 (23.20%)	88 (24.40%)	23 (29.50%)	78 (25.20%)	0.750
Lipid-lowering drugs	6 (3.70%)	2 (0.60%)	3 (3.80%)	5 (1.60%)	0.037
STEMI, n (%)	78 (47.60%)	193 (53.50%)	35 (44.90%)	165 (53.40%)	0.336
Killip ≥ II class, n (%)	1899 (72.10%)	1326 (76.80%)	573 (63.20%)	573 (63.20%)	< 0.001

Data were expressed as mean ± SD, median (interquartile range), or n (%). Group 1: Lp(a) < 30 mg/dL & Hs-CRP < 2 mg/L; Group 2: Lp(a) < 30 mg/dL & Hs-CRP ≥ 2 mg/L; Group 3: Lp(a) ≥ 30 mg/dL & Hs-CRP < 2 mg/L; Group 4: Lp(a) ≥ 30 mg/dL & Hs-CRP ≥ 2 mg/L. Lp(a), lipoprotein(a); Hs-CRP, high-sensitivity C-reactive protein; CHD, coronary heart disease; CKD, chronic kidney disease; STEMI, ST elevation myocardial infarction.

**Table 2:** Biomarkers of patients stratified by Lp(a) and Hs-CRP categories.

	Group 1	Group 2	Group 3	Group 4	P value
LVEF, %	60.10 ± 11.16	55.99 ± 11.70	57.37 ± 12.18	53.32 ± 13.51	< 0.001
BMI, kg/m^2^	24.81 ± 3.22	25.02 ± 3.63	24.74 ± 3.69	24.65 ± 3.54	0.663
SBP, mmHg	133.90 ± 21.62	128.55 ± 21.43	133.81 ± 20.72	132.18 ± 24.22	0.029
DBP, mmHg	80.26 ± 13.70	77.40 ± 13.00	79.69 ± 12.60	77.01 ± 15.87	0.058
Heart rate, bpm	77.99 ± 14.62	82.74 ± 16.42	79.40 ± 13.32	83.91 ± 17.17	0.001
WBC, x10^9^/L	9.31 ± 3.43	11.30 ± 4.29	9.61 ± 3.67	10.74 ± 4.25	< 0.001
Hemoglobin, g/L	144.01 ± 18.70	136.98 ± 23.20	137.73 ± 22.71	131.82 ± 22.93	< 0.001
Platelet, x10^9^/L	207.41 ± 66.57	215.51 ± 67.19	216.32 ± 54.95	225.80 ± 75.87	0.042
Albumin, g/L	39.46 ± 3.61	37.34 ± 4.05	38.95 ± 4.50	36.69 ± 4.56	< 0.001
Triglyceride, mmol/L	1.49 (1.09, 2.08)	1.38 (1.06, 1.92)	1.37 (0.98, 2.28)	1.48 (0.98, 2.06)	0.668
Total cholesterol, mmol/L	4.55 ± 1.19	4.42 ± 1.09	4.72 ± 1.05	4.72 ± 1.30	0.008
LDL-C, mmol/L	2.72 ± 0.89	2.68 ± 0.79	2.93 ± 0.84	2.99 ± 1.00	< 0.001
HDL-C, mmol/L	1.16 ± 0.27	1.11 ± 0.25	1.16 ± 0.21	1.10 ± 0.26	0.035
Apolipoprotein A1, g/L	1.12 ± 0.24	1.02 ± 0.23	1.11 ± 0.19	0.99 ± 0.23	< 0.001
Apoprotein B, g/L	0.86 ± 0.24	0.83 ± 0.22	0.88 ± 0.21	0.91 ± 0.29	0.001
eGFR, ml/min/1.73m^2^	101.96 ± 36.91	86.74 ± 39.14	87.77 ± 35.03	77.60 ± 36.15	< 0.001
Uric acid, umol/L	348.01 ± 101.11	358.98 ± 113.99	335.55 ± 112.93	381.03 ± 140.94	0.004
Fasting blood glucose, mmol/L	6.04 (5.19, 7.72)	6.38 (5.40, 8.16)	5.91 (5.23, 7.11)	6.55 (5.46, 8.48)	0.018
Hemoglobin Alc, %	6.40 ± 1.37	6.80 ± 1.82	6.43 ± 1.44	7.01 ± 1.82	0.012
Fibrinogen, g/L	3.32 ± 0.80	3.95 ± 0.90	3.68 ± 0.71	4.35 ± 0.96	< 0.001
GRACE score	106.44 ± 24.62	121.09 ± 32.00	121.70 ± 34.34	137.61 ± 36.02	< 0.001

Data were expressed as mean ± SD, median (interquartile range), or n (%). Lp(a), lipoprotein(a); Hs-CRP, high-sensitivity C-reactive protein; LVEF, left ventricular ejection fraction; BMI, body mass index; SBP, systolic blood pressure; DBP, diastolic blood pressure; WBC, white blood count; LDL-C, low-density lipoprotein cholesterol; HDL-C, high-density lipoprotein cholesterol; eGFR, estimated glomerular filtration rate.

**Table 3:** Coronary angiography, medication, and outcome data of patients stratified by Lp(a) and Hs-CRP categories.

	Group 1	Group 2	Group 3	Group 4	P value
Coronary angiography, n (%)					
Left main disease	13 (7.90%)	28 (7.80%)	10 (12.80%)	43 (13.90%)	0.038
Three-vessel disease	75 (45.70%)	192 (53.20%)	51 (65.40%)	212 (68.60%)	< 0.001
Multiple vessel disease	132 (80.50%)	295 (81.70%)	67 (85.90%)	276 (89.30%)	0.021
Number of diseased vessels	2.26 ± 0.77	2.35 ± 0.77	2.51 ± 0.73	2.58 ± 0.68	< 0.001
Gensini score	68.01 ± 35.49	74.68 ± 40.16	83.27 ± 44.82	95.17 ± 44.15	< 0.001
PCI/CABG	144 (87.80%)	311 (86.10%)	71 (91.00%)	270 (87.40%)	0.695
Number of stent	1.00 (1.00, 2.00)	1.00 (1.00, 2.00)	1.00 (1.00, 2.00)	1.00 (1.00, 2.00)	0.015
Stent length, mm	30.00 (18.00, 46.50)	28.00 (18.00, 48.00)	33.00 (23.00, 57.75)	33.00 (21.00, 60.00)	0.003
Discharge medication, n (%)					
Aspirin	158 (96.30%)	334 (92.50%)	72 (92.30%)	285 (92.20%)	0.347
Clopidogrel	46 (28.00%)	147 (40.70%)	30 (38.50%)	127 (41.10%)	0.027
Ticagrelor	117 (71.30%)	213 (59.00%)	47 (60.30%)	176 (57.00%)	0.018
Statin	158 (96.30%)	347 (96.10%)	76 (97.40%)	297 (96.10%)	0.952
PCSK9i	34 (20.70%)	39 (10.80%)	8 (10.30%)	31 (10.00%)	0.004
Beta blocker	130 (79.30%)	291 (80.60%)	60 (76.90%)	250 (80.90%)	0.861
ACEI/ARB	97 (59.10%)	195 (54.00%)	47 (60.30%)	174 (56.30%)	0.615
Calcium channel blocker	19 (11.60%)	53 (14.70%)	14 (17.90%)	56 (18.10%)	0.254
Insulin	22 (13.40%)	51 (14.10%)	11 (14.10%)	52 (16.80%)	0.706
Oral hypoglycemic drugs	39 (23.80%)	73 (20.20%)	18 (23.10%)	62 (20.10%)	0.739
Outcomes, n (%)					
All-cause death	16 (9.80%)	77 (21.30%)	13 (16.70%)	111 (35.90%)	< 0.001
Cardiovascular death	7 (4.30%)	32 (8.90%)	12 (15.40%)	86 (27.80%)	< 0.001

Data were expressed as mean ± SD, median (interquartile range), or n (%). Lp(a), lipoprotein(a); Hs-CRP, high-sensitivity C-reactive protein; PCI, percutaneous coronary intervention; CABG, coronary artery bypass grafting; PCSK9i, proprotein convertase subtilisin/kexin type 9 inhibitors; ACEI, angiotensin converting enzyme inhibitors; ARB, angiotensin receptor blocker.

**Figure 2:**
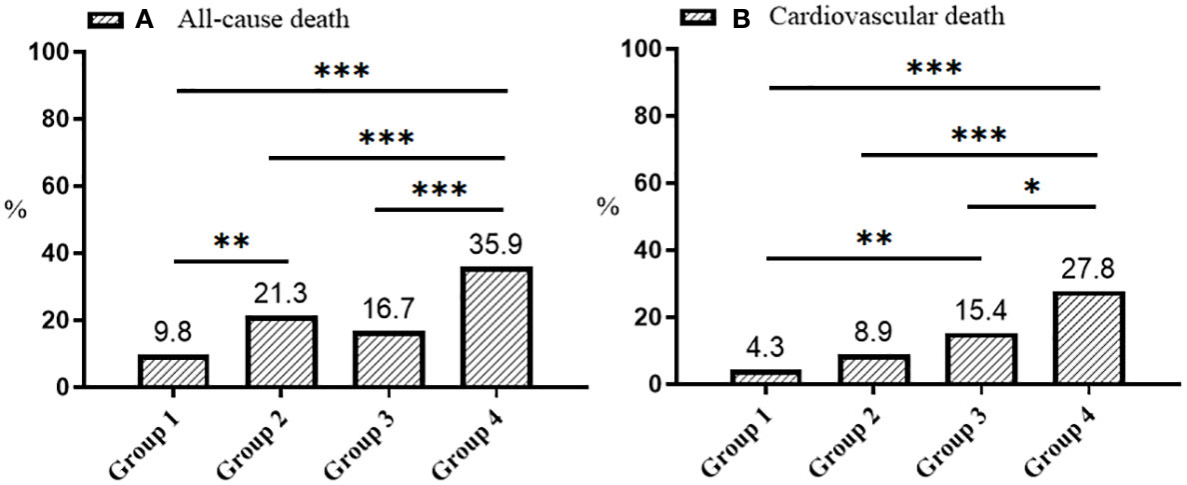
Distribution plots of all-cause **(A)** and cardiovascular death **(B)**. Group 1: Lp(a) < 30 mg/dL & Hs-CRP < 2 mg/L; Group 2: Lp(a) < 30 mg/dL & Hs-CRP ≥ 2 mg/L; Group 3: Lp(a) ≥ 30 mg/dL & Hs-CRP < 2 mg/L; Group 4: Lp(a) ≥ 30 mg/dL & Hs-CRP ≥ 2 mg/L. Lp(a), lipoprotein(a); Hs-CRP, high-sensitivity C-reactive protein; *p < 0.05, **p < 0.01, ***p < 0.001.

### 3.2 Univariate Cox regression analysis of all-cause and cardiovascular death

**Supplementary Table S2** presented the results of univariate Cox regression analysis, demonstrating that factors such as age, sex (male), smoking status, diabetes, hypertension, hyperlipidemia, history of stroke, CKD, use of hypotensive and hypoglycemic drugs, STEMI, Killip class II or higher, LVEF, BMI, DBP, levels of hemoglobin, albumin, ApoA1, ApoB, eGFR, uric acid, FBG, HbA1c, FIB, GRACE score, presence of three-vessel or multiple vessel disease, the number of diseased vessels, Gensini score, PCI/CABG, the number and length of stents, use of CCB, and insulin were all significantly associated with the risk of all-cause death (P < 0.05). Similarly, age, sex (male), diabetes, hypertension, stroke, CKD, use of hypotensive and hypoglycemic drugs, Killip class II or higher, LVEF, DBP, WBC, hemoglobin, albumin, LDL-C, ApoB, eGFR, uric acid, FBG, HbA1c, FIB, GRACE score, left main disease, three-vessel or multiple vessel disease, the number of diseased vessels, Gensini score, PCI/CABG, and insulin use were significantly associated with the risk of cardiovascular death (P < 0.05).

### 3.3 Association between Lp(a) and Hs-CRP with death

**Figure 3:**
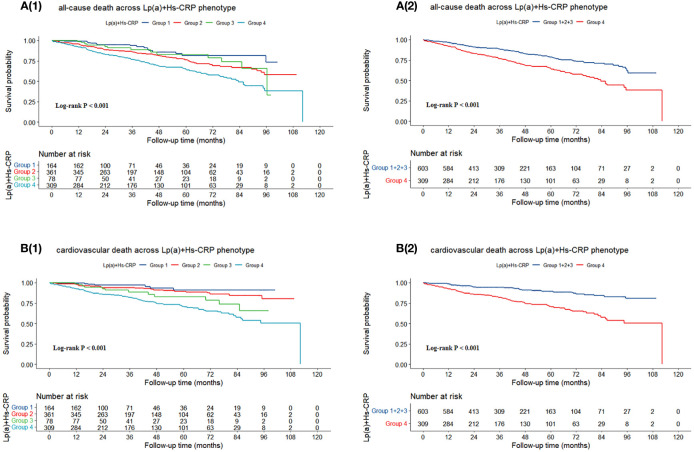
Kaplan-Meier survival curves for **(A)** all-cause death and **(B)** cardiovascular death. Group 1: Lp(a) < 30 mg/dL & Hs-CRP < 2 mg/L; Group 2: Lp(a) < 30 mg/dL & Hs-CRP ≥ 2 mg/L; Group 3: Lp(a) ≥ 30 mg/dL & Hs-CRP < 2 mg/L; Group 4: Lp(a) ≥ 30 mg/dL & Hs-CRP ≥ 2 mg/L. Lp(a), lipoprotein (a); Hs-CRP, high-sensitivity C-reactive protein.

**Figure 3** illustrated that the probability of survival without succumbing to either all-cause mortality or cardiovascular death diminishes as follow-up duration extends, irrespective of the patient categorization into either four or two groups. Notably, this decline was most pronounced among patients with Lp(a) levels ≥ 30 mg/dL and Hs-CRP levels ≥ 2 mg/L (P < 0.001).

The multivariate Cox regression analysis presented in **Table 4** revealed that in the comprehensively adjusted Model 3, only Lp(a) and the combination of Lp(a) and Hs-CRP exhibited a strong association with cardiovascular death risk. Specifically, for Lp(a) levels ≥ 30 mg/dL compared to < 30 mg/dL, the hazard ratio (HR) was 2.434 with a 95% confidence interval (CI) of 1.653–3.583 (P < 0.001); for log_10_(Lp(a)), the HR was 2.630 with a 95% CI of 1.530–4.523 (P < 0.001); for Group 4 versus Group 1, the HR was 2.346 with a 95% CI of 1.054–5.220 (P = 0.037); and for Group 4 versus Groups 1 + 2 + 3, the HR was 1.878 with a 95% CI of 1.284–2.748 (P = 0.001).

**Table 4:** Multivariate Cox regression analysis of all-cause and cardiovascular death.

	Model 1		Model 2		Model 3	
	HR (95% CI)	P value	HR (95% CI)	P value	HR (95% CI)	P value
All-cause death						
Lp(a) < 30 mg/dL	Ref		Ref		Ref	
Lp(a) ≥ 30 mg/dL	1.744 (1.332, 2.285)	< 0.001	1.495 (1.140, 1.959)	0.004	1.175 (0.858, 1.608)	0.316
Log_10_(Lp(a))	2.073 (1.454, 2.956)	< 0.001	1.648 (1.156, 2.349)	0.006	1.163 (0.784, 1.726)	0.453
Hs-CRP < 2 mg/L	Ref		Ref		Ref	
Hs-CRP ≥ 2 mg/L	2.117 (1.431, 3.131)	< 0.001	1.772 (1.196, 2.626)	0.004	1.128 (0.732, 1.740)	0.585
Log_10_(Hs-CRP)	1.704 (1.407, 2.064)	< 0.001	1.553 (1.264, 1.860)	< 0.001	1.176 (0.932, 1.484)	0.171
Lp(a) < 30 mg/dL & Hs-CRP < 2 mg/L	Ref		Ref		Ref	
Lp(a) < 30 mg/dL & Hs-CRP ≥ 2 mg/L	1.936 (1.129, 3.317)	0.016	1.615 (0.941, 2.773)	0.082	1.096 (0.619, 1.940)	0.754
Lp(a) ≥ 30 mg/dL & Hs-CRP < 2 mg/L	1.576 (0.758, 3.277)	0.224	1.312 (0.630, 2.730)	0.468	1.115 (0.514, 2.421)	0.782
Lp(a) ≥ 30 mg/dL & Hs-CRP ≥ 2 mg/L	3.233 (1.912, 5.467)	< 0.001	2.380 (1.403, 4.037)	0.001	1.297 (0.721, 2.333)	0.386
P for trend		< 0.001		0.001		0.725
Other groups	Ref		Ref		Ref	
Lp(a) ≥ 30 mg/dL & Hs-CRP ≥ 2 mg/L	1.952 (1.494, 2.550)	< 0.001	1.648 (1.260, 2.156)	< 0.001	1.194 (0.870, 1.638)	0.272
Cardiovascular death						
Lp(a) < 30 mg/dL	Ref		Ref		Ref	
Lp(a) ≥ 30 mg/dL	3.268 (2.253, 4.741)	< 0.001	2.896 (1.994, 4.205)	< 0.001	2.434 (1.653, 3.583)	< 0.001
Log_10_(Lp(a))	4.978 (3.026, 8.188)	< 0.001	4.114 (2.500, 6.771)	< 0.001	2.630 (1.530, 4.523)	< 0.001
Hs-CRP < 2 mg/L	Ref		Ref		Ref	
Hs-CRP ≥ 2 mg/L	2.032 (1.251, 3.300)	0.004	1.748 (1.074, 2.846)	0.025	0.902 (0.523, 1.557)	0.712
Log_10_(Hs-CRP)	1.703 (1.339, 2.165)	< 0.001	1.559 (1.223, 1.987)	< 0.001	0.951 (0.698, 1.296)	0.751
Lp(a) < 30 mg/dL & Hs-CRP < 2 mg/L	Ref		Ref		Ref	
Lp(a) < 30 mg/dL & Hs-CRP ≥ 2 mg/L	1.846 (0.814, 4.183)	0.142	1.601 (0.706, 3.634)	0.260	0.947 (0.408, 2.197)	0.899
Lp(a) ≥ 30 mg/dL & Hs-CRP < 2 mg/L	3.293 (1.296, 8.367)	0.012	2.878 (1.132, 7.318)	0.026	2.187 (0.839, 5.703)	0.109
Lp(a) ≥ 30 mg/dL & Hs-CRP ≥ 2 mg/L	5.716 (2.643, 12.361)	< 0.001	4.486 (2.068, 9.733)	< 0.001	2.346 (1.054, 5.220)	0.037
P for trend		< 0.001		< 0.001		< 0.001
Other groups	Ref		Ref		Ref	
Lp(a) ≥ 30 mg/dL & Hs-CRP ≥ 2 mg/L	3.134 (2.214, 4.437)	< 0.001	2.736 (1.929, 3.879)	< 0.001	1.878 (1.284, 2.748)	0.001

Model 1: unadjusted; Model 2: adjusted for age and sex; Model 3: adjusted for variables with P < 0.05 in **Table 3** for all-cause death; adjusted for variables with P < 0.05 in **Table 3** for cardiovascular death. Lp(a), lipoprotein (a); Hs-CRP, high-sensitivity C-reactive protein; HR, hazard ratio; CI, confidence interval.

Sensitivity analysis detailed in **Supplementary Table S3**, employing multivariate Cox regression, indicated that post-exclusion of patients with eGFR < 30 ml/min/1.73m^2^, only log_10_(Hs-CRP) was linked to an elevated risk of all-cause death in the fully-adjusted model (HR: 1.471, 95% CI: 1.173–1.844, P = 0.001). Conversely, both Lp(a) and the synergy between Lp(a) and Hs-CRP continued to be independently associated with the risk of cardiovascular death. For Lp(a) ≥ 30 mg/dL versus < 30 mg/dL, the HR was 2.889 with a 95% CI of 1.859–4.489 (P < 0.001); for log_10_(Lp(a)), the HR was 3.910 with a 95% CI of 2.174–7.033 (P < 0.001); for Group 3 versus Group 1, the HR was 3.353 with a 95% CI of 1.133–9.917 (P = 0.029); for Group 4 versus Group 1, the HR was 3.710 with a 95% CI of 1.466–9.392 (P = 0.006); and for Group 4 versus Groups 1 + 2 + 3, the HR was 2.433 with a 95% CI of 1.620–3.656 (P < 0.001).

## 4 Discussion

In this study of hospitalized AMI patients, we found higher baseline Lp(a) and Hs-CRP levels were linked to increased rates of all-cause or cardiovascular death. By grouping patients according to Lp(a) ≥ 30 mg/dL and Hs-CRP ≥ 2 mg/L thresholds, it was revealed that those meeting both criteria had a significantly higher cardiovascular death risk, a finding that was further intensified upon excluding patients with eGFR < 30 ml/min/1.73m^2^. This highlights the critical need to consider the combined impact of these risk factors in managing AMI patient, as their synergy predicts more severe cardiovascular outcomes than either factor alone.

Despite significant advancements in CHD treatment and control of traditional risk factors, adverse prognoses in CHD patients remain common, hinting at underlying residual cardiovascular risks. Lp(a) has emerged as a key player in these residual risks, with evidence linking elevated Lp(a) levels to poorer CHD outcomes (4, 22). Recent clinical trials have further shown that reducing high Lp(a) levels can decrease the risks of cardiovascular event (23, 24), suggesting that targeting Lp(a) could enhance current CHD secondary prevention strategies. However, as Lp(a)-lowering interventions are still under investigation, the necessity to further explore the role of Lp(a) in high-risk groups like AMI patients continues.

Evidence indicates inflammation as another critical residual cardiovascular risk, with studies highlighting its role in atherosclerosis and its association with worse CHD outcomes (4, 25, 26). Therefore, in addition to guideline-directed lifestyle changes and optimal pharmacological treatment aimed at reducing LDL-C levels, pharmacological intervention targeting inflammation could provide further assistance in preventing future cardiac events (27). In recent years, anti-inflammatory treatments that lower Hs-CRP levels have been demonstrated to reduce cardiovascular event risks (28), positioning Hs-CRP as a key inflammatory marker. While Hs-CRP is linked to mortality risks (12), our study did not observe this correlation in AMI patients, suggesting the need for further investigation into its impact on high-risk groups.

Lp(a) has been implicated as a potential acute phase reactant, with its oxidized phospholipids possibly triggering inflammatory responses via interaction with immune cell receptors (29, 30). The LPA gene, containing an IL-6 response element, suggests a pathway where IL-6 inhibition could lower Lp(a) and Hs-CRP levels, thereby reducing cardiovascular risk (31, 32). This indicates a need to investigate whether CHD patients with elevated Lp(a) and Hs-CRP levels could particularly benefit from IL-6 reduction strategies. Moreover, the interplay between Lp(a) and Hs-CRP might exacerbate vascular endothelial atherosclerosis and cardiovascular events through systemic inflammation (4). However, the synergistic effect of Lp(a) and Hs-CRP on cardiovascular risk remains underexplored. Some studies report an increased risk of IHD and MI with high Lp(a) levels in individuals with CRP ≥ 2mg/L, although findings are not consistently significant (33–36). Large cohort studies have provided mixed results on the association between Lp(a), Hs-CRP, with cardiovascular risk, suggesting variability by race, sex, and specific cardiovascular outcomes (37–41). Notably, in certain populations, elevated Lp(a) levels combined with high Hs-CRP significantly correlate with increased risks of major adverse cardiovascular events (MACE), heart failure rehospitalization, and cardiovascular death, while in other populations, such associations have not been observed (38–41). Furthermore, adding other biomarkers like residual cholesterol or D-dimer to the Lp(a) and Hs-CRP combination has shown that the highest levels of these markers correlate with the greatest risk of adverse outcomes in CHD patients (42, 43). Yet, the predictive value of combining Lp(a) and Hs-CRP for death risk in AMI patients remains uncertain. Our study, following 912 AMI patients over 38.98 months, found that those with Lp(a) ≥ 30 mg/dL and Hs-CRP ≥ 2 mg/L had a significantly increased risk of cardiovascular death compared to those with lower levels or other combinations, though no association was found with all-cause death. This suggests that combining multiple biomarkers may better identify cardiovascular event risks than single indicators alone. However, inconsistencies across studies and the impact of population heterogeneity and grouping criteria on outcomes highlight the need for further large-scale prospective research to clarify the joint effects of Lp(a) and Hs-CRP on cardiovascular events and mortality.

Our study highlights the complex interplay between Lp(a) and Hs-CRP in cardiovascular risk. However, limitations include the static baseline measurement of Lp(a) and Hs-CRP, which does not reflect long-term exposure, and the arbitrary threshold-based grouping, which might not capture nuanced associations. Additionally, the single-center design and lack of genetic data limit causal inferences. Changes in medication during follow-up and the unexamined impact of Lp(a) subtypes on inflammatory responses further complicate the interpretation. Future research should dynamically monitor these biomarkers, explore different grouping strategies, and consider genetic factors to elucidate the mechanisms linking Lp(a) and Hs-CRP to cardiovascular outcomes. Furthermore, although this study has revealed the synergistic effect of Lp(a) and Hs-CRP on the risk of all-cause and cardiovascular mortality in AMI patients, Hs-CRP may indicate the need for anti-inflammatory medications such as colchicine. However, these drugs do not affect Lp(a) levels, and therefore, the simultaneous assessment of both does not directly influence treatment choices. Moreover, proposing an algorithm based on Lp(a) and Hs-CRP could more effectively integrate the data from these biomarkers to guide clinical decision-making. Nevertheless, due to resource constraints and aspects of the study design, this study is currently unable to propose such an algorithm. Based on the current findings, future research could explore developing an integrated assessment algorithm that includes both biomarkers to optimize the treatment and management of AMI patients. Finally, some studies suggest setting a higher cutoff value for Lp(a), such as 50 mg/dL. We also acknowledge that there are indeed differences in the cutoff values for Lp(a) across studies, which may stem from factors such as the ethnic backgrounds of the populations studied, pathological states, and research designs. In our study, the choice of a 30 mg/dL cutoff for Lp(a) is based on a review of previous literature and a consensus on risk thresholds applicable to the Chinese population. The participants in this study come from clinical medical centers in China, hence we selected a cutoff value of 30 mg/dL for Lp(a) to more accurately assess its predictive value for all-cause and cardiovascular mortality among Chinese AMI patients. However, considering the international differences in perspectives on higher risk thresholds for Lp(a), this also represents a limitation of the study, restricting the generalizability and applicability of our findings. And Lp(a) levels can significantly vary across different ethnicities, which may influence the assessment of cardiovascular disease risk. Future research could benefit from a more diverse cohort that includes various ethnicities to better understand the ethnic-specific impacts of Lp(a) levels on cardiovascular risk. This would enhance the applicability and relevance of Lp(a) cutoff values across different populations.

## 5 Conclusions

In this real-world cohort study, we did not observe a synergistic effect of Lp(a) and Hs-CRP on all-cause death but uniquely demonstrated that their combination more effectively identifies a higher risk of cardiovascular death than either marker alone. This underscores the importance of considering the synergistic impacts of multiple risk factors on cardiovascular outcomes in clinical settings. Our findings offer a fresh perspective and a theoretical foundation for investigating the combined effects of biomarkers on health. Future research should delve deeper into the mechanisms of Lp(a) and Hs-CRP behind the interaction in AMI patient prognosis.

## Data availability statement

The original contributions presented in the study are included in the article/**Supplementary Material**. Further inquiries can be directed to the corresponding author/s.

## Ethics statement

The studies involving humans were approved by Clinical Research Ethics Committee of Zhongda Hospital affiliated to Southeast University. The studies were conducted in accordance with the local legislation and institutional requirements. The participants provided their written informed consent to participate in this study.

## Author contributions

ZW: Conceptualization, Data curation, Formal analysis, Investigation, Methodology, Software, Visualization, Writing – original draft, Writing – review & editing. JT: Data curation, Formal analysis, Investigation, Visualization, Writing – original draft, Writing – review & editing. QS: Data curation, Formal analysis, Investigation, Visualization, Writing – original draft, Writing – review & editing. LF: Conceptualization, Funding acquisition, Project administration, Supervision, Validation, Writing – review & editing. NL: Conceptualization, Funding acquisition, Project administration, Supervision, Validation, Writing – review & editing. JZ: Conceptualization, Funding acquisition, Project administration, Supervision, Validation, Writing – review & editing.

## References

[1] Roth GA Mensah GA Johnson CO Addolorato G Ammirati E Baddour LM. Global Burden of Cardiovascular Diseases and Risk Factors, 1990–2019: Update From the GBD 2019 Study [published correction appears in J Am Coll Cardiol. 2021 Apr 20;77(15):1958–1959]. J Am Coll Cardiol. (2020) 76:2982–3021. doi: 10.1016/j.jacc.2020.11.010 33309175 PMC7755038

[2] Li Y Zhang J. Disease burden and risk factors of ischemic heart disease in China during 1990–2019 based on the Global Burden of Disease 2019 report: A systematic analysis. Front Public Health. (2022) 10:973317. doi: 10.3389/fpubh.2022.973317 36408039 PMC9670122

[3] Domenico T Rita A Giacomo S Diego A Thelma P Mariana G. Salivary biomarkers for diagnosis of acute myocardial infarction: A systematic review. Int J Cardiol. (2023) 371:54–64. doi: 10.1016/j.ijcard.2022.09.043 36167219

[4] Hoogeveen RC Ballantyne CM. Residual cardiovascular risk at low LDL: remnants, lipoprotein(a), and inflammation. Clin Chem. (2021) 67:143–53. doi: 10.1093/clinchem/hvaa252 PMC7793228 33257928

[5] Ajala ON Everett BM. Targeting inflammation to reduce residual cardiovascular risk. Curr Atheroscler Rep. (2020) 22:66. doi: 10.1007/s11883-020-00883-3 32880743

[6] Hafiane A Daskalopoulou SS. Targeting the residual cardiovascular risk by specific anti-inflammatory interventions as a therapeutic strategy in atherosclerosis. Pharmacol Res. (2022) 178:106157. doi: 10.1016/j.phrs.2022.106157 35257900

[7] Schmidt K Noureen A Kronenberg F Utermann G. Structure, function, and genetics of lipoprotein (a). J Lipid Res. (2016) 57:1339–59. doi: 10.1194/jlr.R067314 PMC4959873 27074913

[8] Reyes-Soffer G Ginsberg HN Berglund L Duell PB Heffron SP Kamstrup PR. Lipoprotein(a): A genetically determined, causal, and prevalent risk factor for atherosclerotic cardiovascular disease: A scientific statement from the American Heart Association. Arterioscler Thromb Vasc Biol. (2022) 42:e48–60. doi: 10.1161/ATV.0000000000000147 PMC9989949 34647487

[9] Sproston NR Ashworth JJ. Role of C-reactive protein at sites of inflammation and infection. Front Immunol. (2018) 9:754. doi: 10.3389/fimmu.2018.00754 29706967 PMC5908901

[10] Yousuf O Mohanty BD Martin SS Joshi PH Blaha MJ Nasir K. High-sensitivity C-reactive protein and cardiovascular disease: a resolute belief or an elusive link? J Am Coll Cardiol. (2013) 62:397–408. doi: 10.1016/j.jacc.2013.05.016 23727085

[11] Kuppa A Tripathi H Al-Darraji A Tarhuni WM Abdel-Latif A. C-reactive protein levels and risk of cardiovascular diseases: A two-sample bidirectional Mendelian randomization study. Int J Mol Sci. (2023) 24:9129. doi: 10.3390/ijms24119129 37298077 PMC10252732

[12] Bernabe-Ortiz A Carrillo-Larco RM Gilman RH Smeeth L Checkley W Miranda JJ. High-sensitivity C-reactive protein and all-cause mortality in four diverse populations: The CRONICAS Cohort Study. Ann Epidemiol. (2022) 67:13–8. doi: 10.1016/j.annepidem.2021.12.007 PMC8960343 34923118

[13] Zhang L He G Huo X Tian A Ji R Pu B. Long-term cumulative high-sensitivity C-reactive protein and mortality among patients with acute heart failure. J Am Heart Assoc. (2023) 12:e029386. doi: 10.1161/JAHA.123.029386 37776214 PMC10727254

[14] Burger PM Pradhan AD Dorresteijn JAN Koudstaal S Teraa M de Borst GJ. C-reactive protein and risk of cardiovascular events and mortality in patients with various cardiovascular disease locations. Am J Cardiol. (2023) 197:13–23. doi: 10.1016/j.amjcard.2023.03.025 37218417

[15] American Diabetes Association. Diagnosis and classification of diabetes mellitus. Diabetes Care. (2013) 36 Suppl 1:S67–74. doi: 10.2337/dc13-S067 PMC3537273 23264425

[16] Rabi DM McBrien KA Sapir-Pichhadze R Nakhla M Ahmed SB Dumanski SM. Hypertension Canada’s 2020 comprehensive guidelines for the prevention, diagnosis, risk assessment, and treatment of hypertension in adults and children. Can J Cardiol. (2020) 36:596–624. doi: 10.1016/j.cjca.2020.02.086 32389335

[17] Joint Committee for the Development of Guidelines for Prevention and Control of Dyslipidaemia in Chinese Adults. Guidelines for prevention and treatment of dyslipidaemia in Chinese adults. Chin J Cardiovasc Dis. (2007) 35:390–419. doi: 10.3760/j.issn:0253-3758.2007.05.003

[18] Webster AC Nagler EV Morton RL Masson P. Chronic kidney disease. Lancet. (2017) 389:1238–52. doi: 10.1016/S0140-6736(16)32064-5 27887750

[19] Ma YC Zuo L Chen JH Luo Q Yu XQ Li Y. Modified glomerular filtration rate estimating equation for Chinese patients with chronic kidney disease [published correction appears in J Am Soc Nephrol. 2006 Dec;17(12):3540]. J Am Soc Nephrol. (2006) 17:2937–44. doi: 10.1681/ASN.2006040368 16988059

[20] Fox KA Fitzgerald G Puymirat E Huang W Carruthers K Simon T. Should patients with acute coronary disease be stratified for management according to their risk? Derivation, external validation and outcomes using the updated GRACE risk score. BMJ Open. (2014) 4:e004425. doi: 10.1136/bmjopen-2013-004425 PMC3931985 24561498

[21] Rampidis GP Benetos G Benz DC Giannopoulos AA Buechel RR. A guide for Gensini Score calculation. Atherosclerosis. (2019) 287:181–3. doi: 10.1016/j.atherosclerosis.2019.05.012 31104809

[22] Sang T Cheng N Dang A Lv N Zhang W Li Y. Lipoprotein (a) is associated with poor long-term prognosis in patients aged 80 years and older with acute coronary syndrome. J Clin Lipidol. (2021) 15:466–76. doi: 10.1016/j.jacl.2021.04.003 34006456

[23] Schwartz GG Szarek M Bittner VA Diaz R Goodman SG Jukema JW. Lipoprotein(a) and benefit of PCSK9 inhibition in patients with nominally controlled LDL cholesterol. J Am Coll Cardiol. (2021) 78:421–33. doi: 10.1016/j.jacc.2021.04.102 PMC8822604 34325831

[24] Bittner VA Szarek M Aylward PE Bhatt DL Diaz R Edelberg JM. Effect of alirocumab on lipoprotein(a) and cardiovascular risk after acute coronary syndrome. J Am Coll Cardiol. (2020) 75:133–44. doi: 10.1016/j.jacc.2019.10.057 31948641

[25] Ridker PM Bhatt DL Pradhan AD Glynn RJ MacFadyen JG Nissen SE. Inflammation and cholesterol as predictors of cardiovascular events among patients receiving statin therapy: a collaborative analysis of three randomised trials. Lancet. (2023) 401:1293–301. doi: 10.1016/S0140-6736(23)00215-5 36893777

[26] Kong P Cui ZY Huang XF Zhang DD Guo RJ Han M. Inflammation and atherosclerosis: signaling pathways and therapeutic intervention. Signal Transduct Target Ther. (2022) 7:131. doi: 10.1038/s41392-022-00955-7 35459215 PMC9033871

[27] Waksman R Merdler I Case BC Waksman O Porto I. Targeting inflammation in atherosclerosis: overview, strategy and directions. EuroIntervention. (2024) 20:32–44. doi: 10.4244/EIJ-D-23-00606 38165117 PMC10756224

[28] Ridker PM Everett BM Thuren T MacFadyen JG Chang WH Ballantyne C. Antiinflammatory therapy with canakinumab for atherosclerotic disease. N Engl J Med. (2017) 377:1119–31. doi: 10.1056/NEJMoa1707914 28845751

[29] Leibundgut G Lee JH Strauss BH Segev A Tsimikas S. Acute and long-term effect of percutaneous coronary intervention on serially-measured oxidative, inflammatory, and coagulation biomarkers in patients with stable angina. J Thromb Thromb. (2016) 41:569–80. doi: 10.1007/s11239-016-1351-6 PMC4811750 26964999

[30] van der Valk FM Bekkering S Kroon J Yeang C Van den Bossche J van Buul JD. Oxidized phospholipids on lipoprotein(a) elicit arterial wall inflammation and an inflammatory monocyte response in humans. Circulation. (2016) 134:611–24. doi: 10.1161/CIRCULATIONAHA.116.020838 PMC4995139 27496857

[31] Ridker PM Devalaraja M Baeres FMM Engelmann MDM Hovingh GK Ivkovic M. IL-6 inhibition with ziltivekimab in patients at high atherosclerotic risk (RESCUE): a double-blind, randomised, placebo-controlled, phase 2 trial. Lancet. (2021) 397:2060–9. doi: 10.1016/S0140-6736(21)00520-1 34015342

[32] Ridker PM Rane M. Interleukin-6 signaling and anti-interleukin-6 therapeutics in cardiovascular disease. Circ Res. (2021) 128:1728–46. doi: 10.1161/CIRCRESAHA.121.319077 33998272

[33] Langsted A Kamstrup PR Nordestgaard BG. Lipoprotein(a): fasting and nonfasting levels, inflammation, and cardiovascular risk. Atherosclerosis. (2014) 234:95–101. doi: 10.1016/j.atherosclerosis.2014.01.049 24632508

[34] Willeit P Kiechl S Kronenberg F Witztum JL Santer P Mayr M. Discrimination and net reclassification of cardiovascular risk with lipoprotein(a): prospective 15-year outcomes in the Bruneck Study [published correction appears in J Am Coll Cardiol. 2016 Feb 16;67(6):737]. J Am Coll Cardiol. (2014) 64:851–60. doi: 10.1016/j.jacc.2014.03.061 25169167

[35] Zhang W Speiser JL Ye F Tsai MY Cainzos-Achirica M Nasir K. High-sensitivity C-reactive protein modifies the cardiovascular risk of lipoprotein(a): multi-ethnic study of atherosclerosis. J Am Coll Cardiol. (2021) 78:1083–94. doi: 10.1016/j.jacc.2021.07.016 PMC8444216 34503676

[36] Colantonio LD Bittner V Safford MM Marcovina S Brown TM Jackson EA. Lipoprotein(a) and the risk for coronary heart disease and ischemic stroke events among black and white adults with cardiovascular disease. J Am Heart Assoc. (2022) 11:e025397. doi: 10.1161/JAHA.121.025397 35621195 PMC9238745

[37] Schwartz GG Szarek M Zeiher A White HD Jukema JW Harrington RA. Elevated C-reactive protein amplifies association of lipoprotein(a) with cardiovascular risk and clinical benefit of alirocumab. J Am Coll Cardiol. (2022) 80:2356–9. doi: 10.1016/j.jacc.2022.09.035 36328873

[38] Wang Y Zhao X Zhou P Liu C Chen R Sheng Z. Impact of postprocedural high-sensitivity C-reactive protein on lipoprotein(a)-associated cardiovascular risk with ST-segment elevation myocardial infarction with percutaneous coronary intervention. Am J Cardiol. (2021) 150:8–14. doi: 10.1016/j.amjcard.2021.03.038 34006374

[39] Puri R Nissen SE Arsenault BJ St John J Riesmeyer JS Ruotolo G. Effect of C-reactive protein on lipoprotein(a)-associated cardiovascular risk in optimally treated patients with high-risk vascular disease: A prespecified secondary analysis of the ACCELERATE trial. JAMA Cardiol. (2020) 5:1136–43. doi: 10.1001/jamacardio.2020.2413 PMC7344788 32639518

[40] Li Z Liu J Shen J Chen Y He L Li M. Association of lipoprotein (a) and 1 year prognosis in patients with heart failure with reduced ejection fraction. ESC Heart Fail. (2022) 9:2399–406. doi: 10.1002/ehf2.13933 PMC9288770 35419980

[41] Yuan D Wang P Jia S Zhang C Zhu P Jiang L. Lipoprotein(a), high-sensitivity C-reactive protein, and cardiovascular risk in patients undergoing percutaneous coronary intervention. Atherosclerosis. (2022) 363:109–16. doi: 10.1016/j.atherosclerosis.2022.10.013 36357218

[42] Li J Zhu P Tang X Jiang L Li Y Yan K. Combined effect of D-dimer, hs-CRP, and Lp(a) on 5-year clinical outcomes after percutaneous coronary intervention: A large real-world study in China. iScience. (2023) 26:107030. doi: 10.1016/j.isci.2023.107030 37485360 PMC10362257

[43] Liu HH Guo YL Zhu CG Wu NQ Gao Y Xu RX. Synergistic effect of the commonest residual risk factors, remnant cholesterol, lipoprotein(a), and inflammation, on prognosis of statin-treated patients with chronic coronary syndrome. J Transl Med. (2022) 20:243. doi: 10.1186/s12967-022-03448-x 35619146 PMC9134647

