# Supplementary material for: Synergistic effect of lipoprotein(a) and high-sensitivity C-reactive protein on the risk of all-cause and cardiovascular death in patients with acute myocardial infarction: a large prospective cohort study
Source: Front Endocrinol (Lausanne). 2024 May 15;15:1392859. doi: 10.3389/fendo.2024.1392859 (PMC11133541; doi:10.3389/fendo.2024.1392859)
Supplement: Supplementary file 1 [file DataSheet_1.docx]

**SUPPLEMENT**

**Table S1.** Baseline characteristics of patients stratified by all-cause and cardiovascular death.

**Table S2.** Univariate Cox regression analysis of all-cause and cardiovascular death.

**Table S3.** Sensitivity analysis: excluding patients with eGFR < 30 ml/min/1.73m^2^.

**Figure S1.** Distribution histogram of Lp(a) and Hs-CRP. Lp(a), lipoprotein(a); Hs-CRP, high-sensitivity C-reactive protein.

| **Table S1.** Baseline characteristics of patients stratified by all-cause and cardiovascular death. | | | | | | | |  |
| --- | --- | --- | --- | --- | --- | --- | --- | --- |
|  |  | All-cause death |  |  | Cardiovascular death |  |  | |
|  | Total population | No | Yes | P value | No | Yes | P value | |
| N | 912 | 695 | 217 |  | 775 | 137 |  | |
| Age, years | 64.66 ± 13.19 | 61.64 ± 12.74 | 74.32 ± 9.44 | < 0.001 | 63.20 ± 13.27 | 72.90 ± 9.13 | < 0.001 | |
| Sex, male, n (%) | 709 (77.70%) | 559 (80.40%) | 150 (69.10%) | < 0.001 | 614 (79.20%) | 95 (69.30%) | 0.010 | |
| Smoking, n (%) | 496 (54.40%) | 404 (58.10%) | 92 (42.40%) | < 0.001 | 431 (55.60%) | 65 (47.40%) | 0.077 | |
| Family history of CHD, n (%) | 110 (12.10%) | 79 (11.40%) | 31 (14.30%) | 0.249 | 90 (11.60%) | 20 (14.60%) | 0.323 | |
| Comorbidities, n (%) |  |  |  |  |  |  |  | |
| Diabetes | 338 (37.10%) | 242 (34.80%) | 96 (44.20%) | 0.012 | 274 (35.40%) | 64 (46.70%) | 0.011 | |
| Hypertension | 661 (72.50%) | 478 (68.80%) | 183 (84.30%) | < 0.001 | 544 (70.20%) | 117 (85.40%) | < 0.001 | |
| Hyperlipidemia | 386 (42.40%) | 308 (44.40%) | 78 (36.10%) | 0.032 | 332 (42.90%) | 54 (39.70%) | 0.488 | |
| Stroke | 228 (25.00%) | 138 (19.90%) | 90 (41.50%) | < 0.001 | 176 (22.70%) | 52 (38.00%) | < 0.001 | |
| CKD | 204 (22.40%) | 97 (14.00%) | 107 (49.30%) | < 0.001 | 132 (17.00%) | 72 (52.60%) | < 0.001 | |
| Treatment, n (%) |  |  |  |  |  |  |  | |
| Hypotensive drugs | 488 (53.50%) | 346 (49.80%) | 142 (65.40%) | < 0.001 | 400 (51.60%) | 88 (64.20%) | 0.006 | |
| Hypoglycemic drugs | 227 (24.90%) | 159 (22.90%) | 68 (31.30%) | 0.012 | 183 (23.60%) | 44 (32.10%) | 0.034 | |
| Lipid-lowering drugs | 16 (1.80%) | 14 (2.00%) | 2 (0.90%) | 0.384 | 14 (1.80%) | 2 (1.50%) | 0.776 | |
| STEMI, n (%) | 471 (51.60%) | 376 (54.10%) | 95 (43.80%) | 0.008 | 405 (52.30%) | 66 (48.20%) | 0.378 | |
| Killip ≥ II class, n (%) | 1899 (72.10%) | 1326 (76.80%) | 573 (63.20%) | < 0.001 | 1326 (76.80%) | 573 (63.20%) | < 0.001 | |
| LVEF, % | 55.95 ± 12.50 | 56.95 ± 12.01 | 52.67 ± 13.48 | < 0.001 | 56.69 ± 12.19 | 51.58 ± 13.43 | < 0.001 | |
| BMI, kg/m^2^ | 24.84 ± 3.53 | 25.05 ± 3.45 | 24.07 ± 3.72 | 0.001 | 24.94 ± 3.52 | 24.20 ± 3.53 | 0.041 | |
| SBP, mmHg | 131.19 ± 22.46 | 130.31 ± 22.59 | 134.01 ± 21.86 | 0.034 | 130.96 ± 22.49 | 132.53 ± 22.37 | 0.451 | |
| DBP, mmHg | 77.98 ± 14.16 | 78.86 ± 14.42 | 75.16 ± 12.92 | 0.001 | 78.65 ± 14.21 | 74.20 ± 13.29 | 0.001 | |
| Heart rate, bpm | 82.00 ± 16.26 | 81.55 ± 15.82 | 83.44 ± 17.57 | 0.157 | 81.68 ± 15.83 | 83.80 ± 18.47 | 0.207 | |
| WBC, x10^9^/L | 10.61 ± 4.15 | 10.60 ± 4.00 | 10.63 ± 4.60 | 0.922 | 10.49 ± 3.94 | 11.26 ± 5.16 | 0.099 | |
| Hemoglobin, g/L | 136.56 ± 22.68 | 140.14 ± 20.55 | 125.10 ± 25.29 | < 0.001 | 138.27 ± 21.91 | 126.90 ± 24.54 | < 0.001 | |
| Platelet, x10^9^/L | 217.61 ± 69.43 | 219.37 ± 68.57 | 211.96 ± 72.01 | 0.170 | 217.24 ± 68.82 | 219.69 ± 73.04 | 0.703 | |
| Albumin, g/L | 37.64 ± 4.32 | 38.35 ± 4.00 | 35.39 ± 4.53 | < 0.001 | 38.02 ± 4.14 | 35.51 ± 4.68 | < 0.001 | |
| Triglyceride, mmol/L | 1.4 (1.0, 2.0) | 1.5 (1.1, 2.1) | 1.3 (1.0, 1.9) | 0.009 | 1.4 (1.0, 2.0) | 1.4 (1.0, 2.0) | 0.447 | |
| Total cholesterol, mmol/L | 4.57 ± 1.19 | 4.56 ± 1.15 | 4.60 ± 1.29 | 0.645 | 4.54 ± 1.15 | 4.73 ± 1.35 | 0.129 | |
| LDL‑C, mmol/L | 2.81 ± 0.90 | 2.79 ± 0.86 | 2.90 ± 1.00 | 0.148 | 2.78 ± 0.87 | 3.01 ± 1.05 | 0.015 | |
| HDL‑C, mmol/L | 1.12 ± 0.26 | 1.13 ± 0.26 | 1.11 ± 0.25 | 0.378 | 1.12 ± 0.26 | 1.13 ± 0.25 | 0.715 | |
| Apolipoprotein A1, g/L | 1.04 ± 0.23 | 1.06 ± 0.23 | 0.98 ± 0.25 | < 0.001 | 1.05 ± 0.23 | 0.99 ± 0.24 | 0.007 | |
| Apoprotein B, g/L | 0.87 ± 0.25 | 0.86 ± 0.24 | 0.89 ± 0.29 | 0.216 | 0.86 ± 0.24 | 0.92 ± 0.31 | 0.031 | |
| Lp(a), mg/dL | 245.0 (110.5, 447.8) | 218.0 (102.0, 399.0) | 336.5 (181.2, 560.5) | < 0.001 | 216.0 (102.8, 399.3) | 421.0 (276.0, 671.0) | < 0.001 | |
| eGFR, ml/min/1.73m^2^ | 86.47 ± 38.27 | 94.14 ± 36.64 | 61.89 ± 32.67 | < 0.001 | 91.30 ± 37.44 | 59.14 ± 30.80 | < 0.001 | |
| Uric acid, umol/L | 362.47 ± 122.39 | 350.25 ± 112.95 | 401.63 ± 142.01 | 0.001 | 352.86 ± 114.15 | 416.84 ± 150.58 | < 0.001 | |
| Fasting blood glucose, mmol/L | 6.3 (5.4, 8.2) | 6.3 (5.4, 7.8) | 6.5 (5.3, 8.9) | 0.454 | 6.3 (5.4, 7.9) | 6.6 (5.3, 9.4) | 0.089 | |
| Hemoglobin Alc, % | 6.75 ± 1.72 | 6.57 ± 1.57 | 7.46 ± 2.08 | < 0.001 | 6.60 ± 1.58 | 7.71 ± 2.17 | < 0.001 | |
| Fibrinogen, g/L | 3.95 ± 0.96 | 3.85 ± 0.95 | 4.26 ± 0.91 | < 0.001 | 3.87 ± 0.95 | 4.34 ± 0.91 | < 0.001 | |
| Hs-CRP, mg/L | 8.1 (1.8, 29.2) | 5.6 (1.3, 22.1) | 16.0 (6.1, 61.5) | < 0.001 | 6.6 (1.5, 24.9) | 16.5 (5.8, 69.7) | < 0.001 | |
| GRACE score | 124.10 ± 34.26 | 116.41 ± 31.57 | 148.75 ± 30.75 | < 0.001 | 119.27 ± 32.33 | 151.46 ± 32.01 | < 0.001 | |
| Coronary angiography, n (%) |  |  |  |  |  |  |  | |
| Left main disease | 94 (10.30%) | 67 (9.60%) | 27 (12.40%) | 0.236 | 72 (9.30%) | 22 (16.10%) | 0.016 | |
| Three-vessel disease | 530 (58.10%) | 373 (53.70%) | 157 (72.40%) | < 0.001 | 426 (55.00%) | 104 (75.90%) | < 0.001 | |
| Multiple vessel disease | 770 (84.40%) | 572 (82.30%) | 198 (91.20%) | 0.002 | 644 (83.10%) | 126 (92.00%) | 0.008 | |
| Number of diseased vessels | 2.43 ± 0.74 | 2.36 ± 0.76 | 2.64 ± 0.63 | < 0.001 | 2.38 ± 0.76 | 2.68 ± 0.62 | < 0.001 | |
| Gensini score | 81.16 ± 42.50 | 78.35 ± 40.79 | 90.13 ± 46.52 | 0.001 | 78.19 ± 40.72 | 97.96 ± 48.25 | < 0.001 | |
| PCI/CABG | 796 (87.30%) | 622 (89.50%) | 174 (80.20%) | < 0.001 | 687 (88.60%) | 109 (79.60%) | 0.003 | |
| Number of stent | 1.0 (1.0, 2.0) | 1.0 (1.0, 2.0) | 1.0 (1.0, 2.0) | 0.007 | 1.0 (1.0, 2.0) | 1.0 (1.0, 2.0) | 0.309 | |
| Stent length, mm | 30.0 (18.0, 52.0) | 30.0 (21.0, 52.0) | 28.0 (18.0, 49.3) | 0.013 | 30.0 (20.0, 52.0) | 29.0 (18.0, 52.0) | 0.318 | |
| Discharge medication, n (%) |  |  |  |  |  |  |  | |
| Aspirin | 849 (93.10%) | 651 (93.70%) | 198 (91.20%) | 0.219 | 725 (93.50%) | 124 (90.50%) | 0.196 | |
| Clopidogrel | 350 (38.40%) | 237 (34.10%) | 113 (52.10%) | < 0.001 | 286 (36.90%) | 64 (46.70%) | 0.029 | |
| Ticagrelor | 553 (60.60%) | 453 (65.20%) | 100 (46.10%) | < 0.001 | 484 (62.50%) | 69 (50.40%) | 0.008 | |
| Statin | 878 (96.30%) | 669 (96.30%) | 209 (96.30%) | 0.971 | 746 (96.30%) | 132 (96.40%) | 0.958 | |
| PCSK9i | 122 (12.30%) | 105 (15.10%) | 7 (3.20%) | < 0.001 | 109 (14.10%) | 3 (2.20%) | < 0.001 | |
| Beta blocker | 731 (80.20%) | 563 (81.00%) | 168 (77.40%) | 0.247 | 628 (81.00%) | 103 (75.20%) | 0.114 | |
| ACEI/ARB | 513 (56.30%) | 391 (56.30%) | 122 (56.20%) | 0.992 | 434 (56.00%) | 79 (57.70%) | 0.717 | |
| Calcium channel blocker | 142 (15.60%) | 95 (13.70%) | 47 (21.70%) | 0.005 | 117 (15.10%) | 25 (18.20%) | 0.348 | |
| Insulin | 136 (14.90%) | 88 (12.70%) | 48 (22.10%) | 0.001 | 101 (13.00%) | 35 (25.50%) | < 0.001 | |
| Oral hypoglycemic drugs | 192 (21.10%) | 144 (20.70%) | 48 (22.10%) | 0.659 | 162 (20.90%) | 30 (21.90%) | 0.792 | |

Data were expressed as mean ± SD, median (interquartile range), or n (%). CHD, coronary heart disease; CKD, chronic kidney disease; STEMI, ST elevation myocardial infarction; LVEF, left ventricular ejection fraction; BMI, body mass index; SBP, systolic blood pressure; DBP, diastolic blood pressure; WBC, white blood count; LDL-C, low-density lipoprotein cholesterol; HDL-C, high-density lipoprotein cholesterol; Lp(a), lipoprotein(a); eGFR, estimated glomerular filtration rate; Hs-CRP, high-sensitivity C-reactive protein; PCI, percutaneous coronary intervention; CABG, coronary artery bypass grafting; PCSK9i, proprotein convertase subtilisin/kexin type 9 inhibitors; ACEI, angiotensin converting enzyme inhibitors; ARB, angiotensin receptor blocker.

| **Table S2.** Univariate Cox regression analysis of all-cause and cardiovascular death. | | | | |
| --- | --- | --- | --- | --- |
|  | All-cause death | | Cardiovascular death | |
|  | HR (95% CI) | P value | HR (95% CI) | P value |
| Age | 1.078 (1.064, 1.093) | < 0.001 | 1.063 (1.047, 1.080) | < 0.001 |
| Male | 0.649 (0.487, 0.867) | 0.003 | 0.653 (0.454, 0.940) | 0.022 |
| Smoking | 0.642 (0.490, 0.842) | 0.001 | 0.786 (0.561, 1.101) | 0.162 |
| Family history of CHD | 0.981 (0.670, 1.437) | 0.923 | 1.016 (0.631, 1.635) | 0.948 |
| Comorbidities |  |  |  |  |
| Diabetes | 1.583 (1.210, 2.071) | 0.001 | 1.742 (1.243, 2.441) | 0.001 |
| Hypertension | 2.253 (1.562, 3.249) | < 0.001 | 2.425 (1.508, 3.897) | < 0.001 |
| Hyperlipidemia | 0.756 (0.573, 0.998) | 0.049 | 0.884 (0.627, 1.248) | 0.483 |
| Stroke | 2.459 (1.875, 3.226) | < 0.001 | 2.080 (1.469, 2.944) | < 0.001 |
| Chronic kidney disease | 3.704 (2.836, 4.837) | < 0.001 | 4.216 (3.010, 5.905) | < 0.001 |
| Treatment |  |  |  |  |
| Hypotensive drugs | 1.965 (1.484, 2.602) | < 0.001 | 1.840 (1.296, 2.613) | 0.001 |
| Hypoglycemic drugs | 1.719 (1.288, 2.293) | < 0.001 | 1.757 (1.226, 2.520) | 0.002 |
| Lipid-lowering drugs | 0.696 (0.173, 2.805) | 0.610 | 1.082 (0.267, 4.379) | 0.912 |
| STEMI | 0.654 (0.500, 0.856) | 0.002 | 0.792 (0.566, 1.110) | 0.176 |
| Killip ≥ II class | 2.781 (2.123, 3.642) | < 0.001 | 3.488 (2.467, 4.931) | < 0.001 |
| Left ventricular ejection fraction | 0.974 (0.963, 0.985) | < 0.001 | 0.967 (0.954, 0.981) | < 0.001 |
| Body mass index | 0.936 (0.894, 0.979) | 0.004 | 0.947 (0.895, 1.002) | 0.059 |
| Systolic blood pressure | 1.005 (0.999, 1.011) | 0.112 | 1.002 (0.994, 1.009) | 0.626 |
| Diastolic blood pressure | 0.982 (0.972, 0.992) | < 0.001 | 0.977 (0.964, 0.989) | < 0.001 |
| Heart rate | 1.007 (1.000, 1.015) | 0.061 | 1.009 (0.999, 1.018) | 0.075 |
| White blood count | 1.010 (0.978, 1.043) | 0.541 | 1.045 (1.006, 1.085) | 0.022 |
| Hemoglobin | 0.978 (0.974, 0.983) | < 0.001 | 0.981 (0.975, 0.987) | < 0.001 |
| Platelet | 0.999 (0.997, 1.001) | 0.417 | 1.001 (0.998, 1.003) | 0.515 |
| Albumin | 0.887 (0.861, 0.913) | < 0.001 | 0.892 (0.860, 0.925) | < 0.001 |
| Triglyceride | 0.870 (0.755, 1.002) | 0.054 | 0.940 (0.805, 1.098) | 0.437 |
| Total cholesterol | 1.040 (0.930, 1.163) | 0.496 | 1.126 (0.988, 1.283) | 0.076 |
| Low-density lipoprotein cholesterol | 1.088 (0.939, 1.260) | 0.261 | 1.232 (1.042, 1.457) | 0.015 |
| High-density lipoprotein cholesterol | 1.112 (0.657, 1.882) | 0.693 | 1.493 (0.783, 2.850) | 0.224 |
| Apolipoprotein A1 | 0.500 (0.271, 0.926) | 0.027 | 0.556 (0.257, 1.200) | 0.135 |
| Apoprotein B | 2.172 (1.284, 3.673) | 0.004 | 3.215 (1.769, 5.841) | < 0.001 |
| eGFR | 0.978 (0.973, 0.982) | < 0.001 | 0.975 (0.969, 0.980) | < 0.001 |
| Uric acid | 1.003 (1.002, 1.004) | < 0.001 | 1.003 (1.002, 1.005) | < 0.001 |
| Fasting blood glucose | 1.032 (1.009, 1.055) | 0.006 | 1.039 (1.015, 1.063) | 0.001 |
| Hemoglobin Alc | 1.231 (1.121, 1.350) | < 0.001 | 1.306 (1.173, 1.455) | < 0.001 |
| Fibrinogen | 1.396 (1.203, 1.619) | < 0.001 | 1.539 (1.286, 1.842) | < 0.001 |
| GRACE score | 1.021 (1.018, 1.025) | < 0.001 | 1.023 (1.018, 1.028) | < 0.001 |
| Coronary angiography |  |  |  |  |
| Left main disease | 1.280 (0.855, 1.917) | 0.230 | 1.712 (1.084, 2.703) | 0.021 |
| Three-vessel disease | 2.012 (1.494, 2.711) | < 0.001 | 2.388 (1.613, 3.536) | < 0.001 |
| Multiple vessel disease | 2.172 (1.356, 3.479) | 0.001 | 2.345 (1.266, 4.345) | 0.007 |
| Number of diseased vessels | 1.662 (1.342, 2.059) | < 0.001 | 1.824 (1.374, 2.422) | < 0.001 |
| Gensini score | 1.006 (1.003, 1.009) | < 0.001 | 1.010 (1.006, 1.014) | < 0.001 |
| PCI/CABG | 0.492 (0.352, 0.687) | < 0.001 | 0.475 (0.313, 0.721) | < 0.001 |
| Number of stent | 0.804 (0.697, 0.927) | 0.003 | 0.889 (0.751, 1.053) | 0.172 |
| Stent length, mm | 0.994 (0.989, 0.999) | 0.022 | 0.998 (0.992, 1.003) | 0.415 |
| Discharge medication |  |  |  |  |
| Aspirin | 0.711 (0.444, 1.139) | 0.156 | 0.656 (0.370, 1.162) | 0.149 |
| Clopidogrel | 1.233 (0.938, 1.621) | 0.133 | 1.013 (0.717, 1.430) | 0.942 |
| Ticagrelor | 0.772 (0.587, 1.014) | 0.063 | 0.898 (0.637, 1.266) | 0.539 |
| Statin | 0.885 (0.436, 1.795) | 0.735 | 0.888 (0.363, 2.172) | 0.795 |
| PCSK9i | 0.581 (0.270, 1.249) | 0.164 | 0.354 (0.111, 1.123) | 0.078 |
| Beta blocker | 0.942 (0.685, 1.296) | 0.712 | 0.823 (0.558, 1.215) | 0.327 |
| ACEI/ARB | 1.008 (0.770, 1.319) | 0.955 | 1.075 (0.765, 1.512) | 0.677 |
| Calcium channel blocker | 1.588 (1.149, 2.195) | 0.005 | 1.272 (0.824, 1.964) | 0.277 |
| Insulin | 1.937 (1.404, 2.671) | < 0.001 | 2.318 (1.577, 3.407) | < 0.001 |
| Oral hypoglycemic drugs | 1.337 (0.969, 1.845) | 0.077 | 1.300 (0.866, 1.952) | 0.205 |

CHD, coronary heart disease; STEMI, ST elevation myocardial infarction; eGFR, estimated glomerular filtration rate; PCI, percutaneous coronary intervention; CABG, coronary artery bypass grafting; PCSK9i, proprotein convertase subtilisin/kexin type 9 inhibitors; ACEI, angiotensin converting enzyme inhibitors; ARB, angiotensin receptor blocker.

| **Table S3.** Sensitivity analysis: excluding patients with eGFR < 30 ml/min/1.73m^2^. | | | | |
| --- | --- | --- | --- | --- |
|  | Model 1 | | Model 2 | |
|  | HR (95% CI) | P value | HR (95% CI) | P value |
| All-cause death |  |  |  |  |
| Lp(a) < 30 mg/dL | Ref |  | Ref |  |
| Lp(a) ≥ 30 mg/dL | 1.527 (1.134, 2.057) | 0.005 | 1.213 (0.855, 1.723) | 0.279 |
| Log_10_(Lp(a)) | 1.636 (1.107, 2.418) | 0.014 | 1.185 (0.771, 1.822) | 0.439 |
| Hs-CRP < 2 mg/L | Ref |  | Ref |  |
| Hs-CRP ≥ 2 mg/L | 1.705 (1.122, 2.590) | 0.012 | 1.218 (0.755, 1.964) | 0.420 |
| Log_10_(Hs-CRP) | 1.560 (1.259, 1.933) | < 0.001 | 1.471 (1.173, 1.844) | 0.001 |
| Lp(a) < 30 mg/dL & Hs-CRP < 2 mg/L | Ref |  | Ref |  |
| Lp(a) < 30 mg/dL & Hs-CRP ≥ 2 mg/L | 1.565 (0.875, 2.802) | 0.131 | 1.209 (0.650, 2.249) | 0.549 |
| Lp(a) ≥ 30 mg/dL & Hs-CRP < 2 mg/L | 1.369 (0.632, 2.965) | 0.426 | 1.191 (0.511, 2.775) | 0.686 |
| Lp(a) ≥ 30 mg/dL & Hs-CRP ≥ 2 mg/L | 2.353 (1.335, 4.149) | 0.003 | 1.475 (0.772, 2.820) | 0.239 |
| P for trend |  | 0.003 |  | 0.603 |
| Other groups | Ref |  | Ref |  |
| Lp(a) ≥ 30 mg/dL & Hs-CRP ≥ 2 mg/L | 1.664 (1.239, 2.234) | 0.001 | 1.252 (0.874, 1.793) | 0.221 |
| Cardiovascular death |  |  |  |  |
| Lp(a) < 30 mg/dL | Ref |  | Ref |  |
| Lp(a) ≥ 30 mg/dL | 3.292 (2.151, 5.038) | < 0.001 | 2.889 (1.859, 4.489) | < 0.001 |
| Log_10_(Lp(a)) | 4.840 (2.751, 8.517) | < 0.001 | 3.910 (2.174, 7.033) | < 0.001 |
| Hs-CRP < 2 mg/L | Ref |  | Ref |  |
| Hs-CRP ≥ 2 mg/L | 1.775 (1.043, 3.022) | 0.034 | 0.955 (0.552, 1.747) | 0.881 |
| Log_10_(Hs-CRP) | 1.641 (1.250, 2.154) | < 0.001 | 1.085 (0.760, 1.549) | 0.652 |
| Lp(a) < 30 mg/dL & Hs-CRP < 2 mg/L | Ref |  | Ref |  |
| Lp(a) < 30 mg/dL & Hs-CRP ≥ 2 mg/L | 1.781 (0.679, 4.674) | 0.241 | 1.340 (0.504, 3.567) | 0.557 |
| Lp(a) ≥ 30 mg/dL & Hs-CRP < 2 mg/L | 3.704 (1.285, 10.674) | 0.015 | 3.353 (1.133, 9.917) | 0.029 |
| Lp(a) ≥ 30 mg/dL & Hs-CRP ≥ 2 mg/L | 5.523 (2.222, 13.731) | < 0.001 | 3.710 (1.466, 9.392) | 0.006 |
| P for trend |  | < 0.001 |  | < 0.001 |
| Other groups | Ref |  | Ref |  |
| Lp(a) ≥ 30 mg/dL & Hs-CRP ≥ 2 mg/L | 2.954 (1.999, 4.363) | < 0.001 | 2.433 (1.620, 3.656) | < 0.001 |

Model 1: adjusted for age and sex. Model 2: adjusted for all covariates used in Model 3 of Table 4. Lp(a), lipoprotein (a); Hs-CRP, high-sensitivity C-reactive protein; HR, hazard ratio; CI, confidence interval.


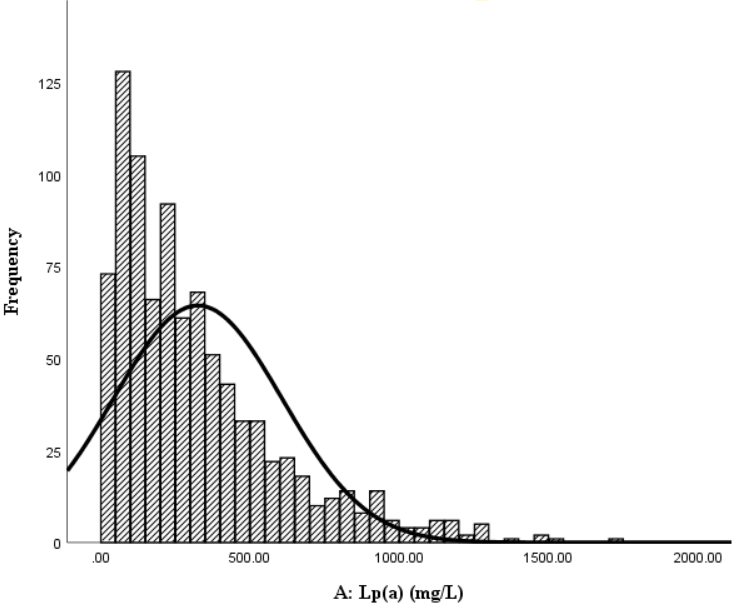

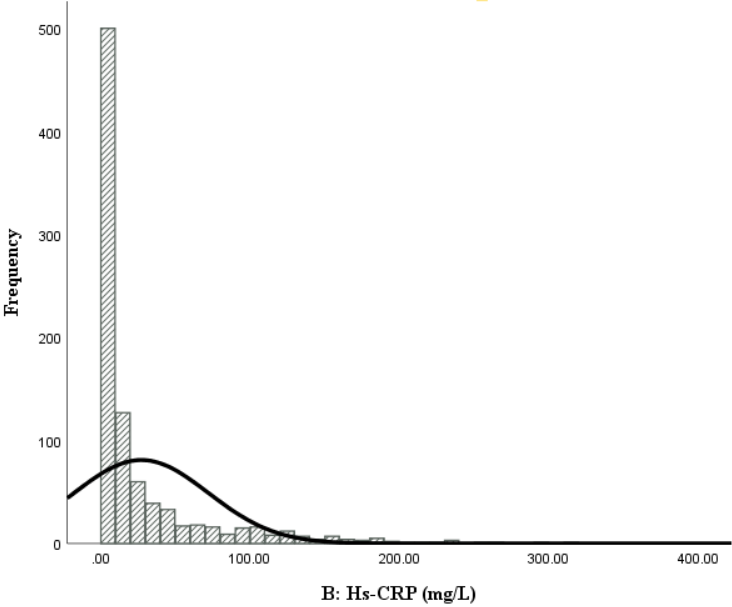


**Figure S1.** Distribution histogram of Lp(a) and Hs-CRP.

Lp(a), lipoprotein(a); Hs-CRP, high-sensitivity C-reactive protein.
